# Systematic Literature Review on ICF From 2001 to 2013 in the Nordic Countries Focusing on Clinical and Rehabilitation Context

**DOI:** 10.14740/jocmr2400w

**Published:** 2015-12-03

**Authors:** Thomas Maribo, Kirsten S. Petersen, Charlotte Handberg, Hanne Melchiorsen, Anne-Mette H. Momsen, Claus V. Nielsen, Matilde Leonardi, Merete Labriola

**Affiliations:** a Section of Social Medicine and Rehabilitation, Department of Public Health, Rehabilitation Center Marselisborg, Aarhus University, Denmark; b Public Health and Quality Improvement, Central Denmark Region, Aarhus, Denmark; c Neurology Public Health and Disability Unit, Neurological Institute C. Besta, IRCCS Foundation, Milan, Italy

**Keywords:** Rehabilitation, Clinical context, Non-clinical context, ICF, Components, Intervention

## Abstract

We present a systematic review on International Classification of Functioning, Disability and Health (ICF) used in the Nordic countries from 2001 through 2013, describing and quantifying the development in utilization of ICF, and describe the extent to which the different components of the ICF have been used. A search was conducted in EMBASE, MEDLINE and PsycInfo. Papers from Nordic countries were included if ICF was mentioned in title or abstract. Papers were assigned to one of eight categories covering the wide rehabilitation area; furthermore, area of focus was assigned. Use of ICF components and intervention were coded in papers categorized as “clinical and/or rehabilitation contexts” or “non-clinical contexts”. One hundred seventy papers were included, of these 99 papers were from the categories “clinical and/or rehabilitation contexts” or “non-clinical contexts”. Forty-two percent of the 170 included papers were published in the period 2011 - 2013. There was an increase in ICF-relevant papers from 2001 to 2013, especially in the categories “clinical and/or rehabilitation contexts” and “non-clinical contexts”. The most represented focus areas were neurology, musculoskeletal, and work-related areas. All five or at least four ICF components were mentioned in the results or discussions in most papers, and activity was most frequently mentioned.

## Introduction

Has the mission been successful, does the International Classification of Functioning, Disability and Health (ICF) provide a common language? The ICF was developed by the World Health Organization (WHO) and formally endorsed in 2001 as a framework to document and evaluate functioning and disability [1]. ICF is a classification system aimed at supporting standardized identification and description of health and health-related stages based on the bio-psychosocial model that defines the interaction between a person with a health condition and his/her environment [1]. In the Nordic countries, ICF has been adopted into clinical, educational and rehabilitation contexts and is used as a framework to guide clinical practice [2, 3].

The overall aim of the ICF is to provide a common language of functioning in order to facilitate data comparisons and a systematic coding scheme for health information systems [1]. The ICF conceptualizes functioning in a holistic framework as comprising body functions, structures, activities and participation, taking into account simultaneous effects of environmental and personal factors (the latter not classified in ICF). Thus, focus is not on cure and illness but on the person’s functioning in his or her actual life and environment; ICF integrates physical, psychological, social and contextual factors of functioning [1, 4, 5]. The ICF has been widely described, discussed and referred to in published literature [4-9], yet further discussion on framework and structure will not be elaborated in this paper, as the objective is to get an overview of the literature.

Since 2001, the ICF has become widely accepted both as a theoretical framework and as a useful clinical practice tool used across different health and social disciplines. In 2009, Jelsma published a literature survey on papers published until 2007 [7] followed by a systematic review including 670 papers in 2011 by Cerniauskaite et al “Systematic literature review on ICF from 2001 to 2009: its use, implementation and operationalisation” concluding that the impact of ICF on rehabilitation and rehabilitation research has been substantial [6]. Thus, they found a positive change in how functioning and disability are used in literature and an increase in the number of published papers, as more than 20% were published in 2009. Of the included papers, 173 (25.9%) were in the category “clinical and/or rehabilitation contexts” and 62 (9.2%) were “non-clinical contexts” [6]. If, however, the ICF is to have true practical application, more papers on ICF in “clinical and rehabilitation contexts” are needed as well as papers on “non-clinical contexts”. In the period 2001 - 2009, almost one-third (30.8%) of the papers were conceptual papers, and as ICF should be well known by now, the number of such papers will presumably decrease over the years [6]. Hopefully, the spread in general knowledge will lead to more papers on the use of ICF in clinical, non-clinical and rehabilitation settings.

There is no consensus in reporting ICF results in the literature, and the quality of the ICF papers within “clinical practice and/or rehabilitation contexts” and “non-clinical contexts” varies a lot. A group under the WHO Family of International Classifications (WHO-FIC) Network - the Functioning and Disability Reference Group (FDRG) - has discussed criteria to evaluate literature related to ICF [10]. These criteria have been discussed between international experts. It is recommended that as a general rule, good quality papers on ICF should have considered at least all components of the ICF framework, and reasons for excluding one or more components should always be explained.

To set the threshold criteria for good quality papers, and in order to explore the quality of papers from the categories “clinical and/or rehabilitation contexts” and “non-clinical contexts”, it is necessary to select a sample of papers between the many hundreds published from 2001 to 2013. Cerniauskaite et al in fact found 173 papers from “clinical and/or rehabilitation contexts”, and this figure would be tripled if papers from 2001 to 2013 were included. Thus, it seems reasonable to apply selection criteria to reduce the scope of the study; therefore we have applied a geographic selection criterion. The Nordic countries (Denmark, Finland, Iceland, Norway and Sweden) are not only geographically close to each other, but also largely similar in terms of their welfare systems, although it is acknowledged that no two welfare states are quite alike. In the Nordic health and welfare systems, strong state intervention, and wide universal welfare schemes are among the key traits. In the Nordic countries, the recommendations on ICF have followed international trends, making the region adequately representative as a focus for study [2, 3].

The objective of this study was to present a systematic review on ICF used in the Nordic countries from its release in 2001 through 2013, describing and quantifying the development in utilization of ICF within the categories “clinical and/or rehabilitation contexts” and “non-clinical context”. Furthermore, the objective was to describe the extent to which the different components of the ICF have been used.

## Methods

### Data sources

A systematic literature search was conducted using three electronic databases, EMBASE, MEDLINE and PsycInfo, in June 2013 and again in May 2014. The search strategy used by Cerniauskaite et al [6] was refined, e.g. the term “disability” was replaced with “disabil*” which caused a marked increase in the amount of hits. Furthermore, ICIDH was included as a keyword as ICF became an MeSH word in PubMed as late as 2012, meaning that some papers from early ICF years with ICIDH as a keyword were discovered. Advanced search methods were used with different combinations of the following keywords: “ICF”, “international classification functioning disabil* health”, “classification functioning”, “classification disabil*”, “classification handicap*”, “classification health”, “classification impairment”, and “ICIDH”. The search strategies used for the three databases varied slightly due to differences in construction. The search was restricted to papers in English.

### Selection

Papers were eligible for inclusion if they met the following inclusion criteria: date of publication from 2001 up to and including 2013; ICF or International Classification of Functioning, Disability and Health mentioned in the title or abstract; the language of the abstract was English; and if the first author was affiliated with a Nordic country. Peer reviewed original and review papers were included, excluding book chapters and theses. Papers were excluded if they only mentioned ICIDH or ICIDH-2, if they were on ICF-CY only, or if they were on persons aged < 18 years only. No additional papers were included. The inclusion was performed by five teams of reviewers; each record was read by two reviewers. In case of disagreement, the record was discussed in the research group until consensus was reached.

### Data extraction

Full texts of all papers included in this review were read independently by two researchers from the research group and assigned to one of six categories, created by Cerniauskaite et al [6]: 1) conceptual papers, 2) development of ICF and of ICF-related instruments, 3) clinical and/or rehabilitation contexts, 4) non-clinical contexts, 5) linking papers, and 6) ICF only mentioned; additionally another two categories were added: 7) reviews and 8) protocols.

Furthermore, an area of focus was assigned to all papers. Area of focus was generated from medical specialities and expanded with relevant areas such as “elderly” and “mixed population”. Editorials papers on the ICF framework were coded “not relevant” as they do not cover a single area of focus. All papers categorized as “clinical and/or rehabilitation contexts” or “non-clinical contexts” were reread with focus on the use of the components (body functions, body structures, activity, participation, environmental factors, and personal factors) mentioned in results or discussion and whether the paper represented an intervention study. Intervention was defined as “a treatment, whether for preventative or therapeutic reasons, an assessment or diagnostic tool or some other type of service or condition to which a patient might be exposed” [11]. Papers featuring intervention were coded to indicate whether intervention was multidisciplinary or mono-disciplinary according to who performed the intervention. Furthermore, the type of analysis (qualitative, quantitative or both) was coded.

In case of disagreement amongst the two reviewers, one of the leaders of the research group (TM or ML) read and categorized the study blinded for previous coding. In case of further disagreement, the final decision was taken after a discussion of the issue.

## Results

**Figure 1:**
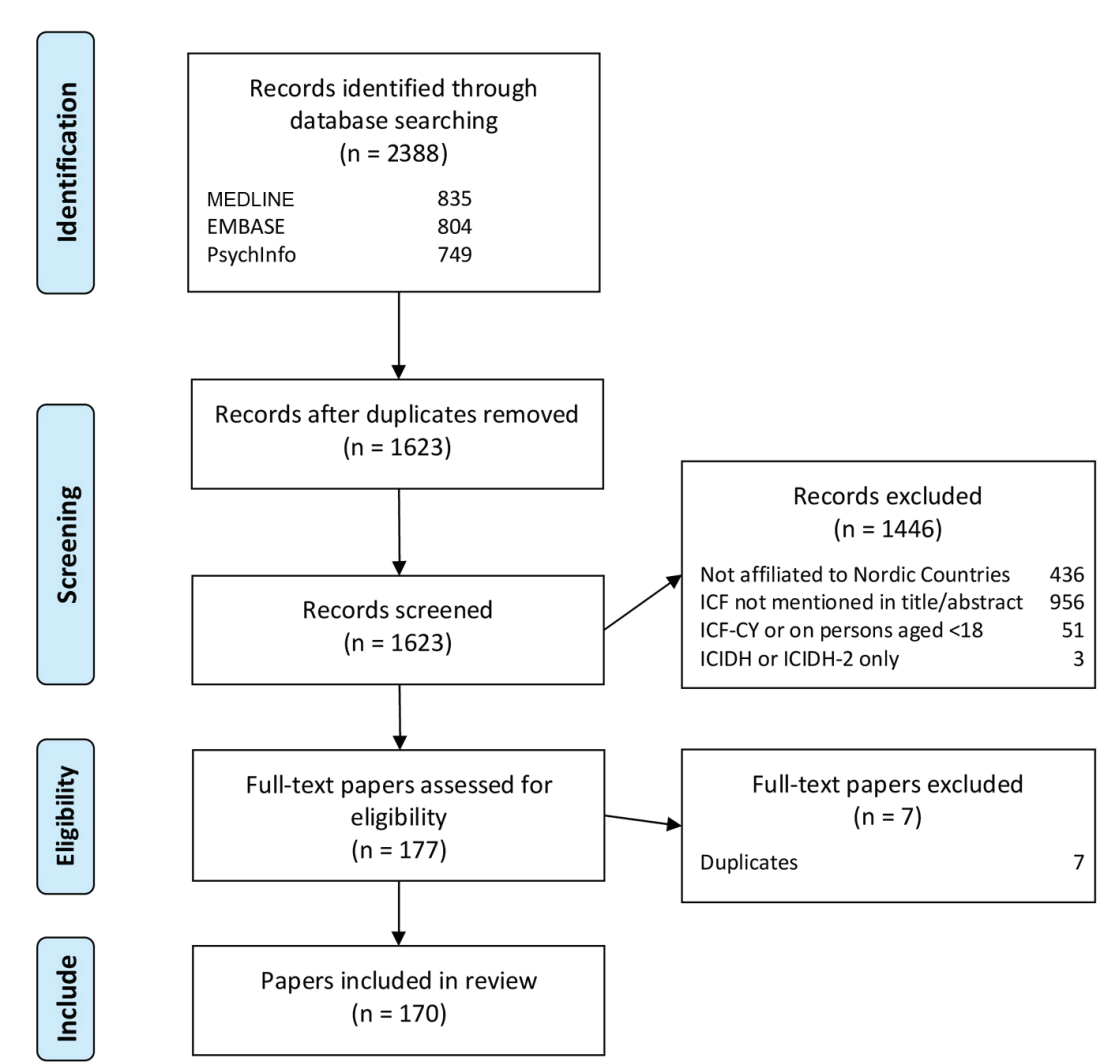
Flow diagram.

Totally 2,388 papers were found, after removing duplicates, 1,623 papers were assessed for eligibility, and finally 170 papers were included in the review. Figure 1 shows the selection process of this systematic review of the literature. A full reference list including the results section can be acquired from the corresponding author.

Information on publication year and the eight categories is reported in Table 1. The majority of papers were categorized as “clinical and/or rehabilitation contexts” (47.6%) and 11.2 % of the papers were in the category “non-clinical context”, making this group the largest with 99 papers (58.8%). There was a marked increase in papers in these categories between 2005 and 2006 and again after 2010. Generally a surge in papers was found in the last 3 years of the inclusion period as 72 (41.6%) of the included papers were published in the period 2011 - 2013.

**Table 1:** Categories and Year of Publication

Categories/year	2002	2003	2004	2005	2006	2007	2008	2009	2010	2011	2012	2013	Total (%)
Clinical and/or rehabilitation contexts	0	1	1	4	4	10	7	10	6	12	14	11	80 (47.6)
Non-clinical contexts	0	0	0	0	3	2	4	1	1	3	3	2	19 (11.2)
Development of ICF and ICF-related instruments	0	0	0	1	3	2	2	4	6	7	5	9	37 (21.8)
Comments and editorials	1	1	1	1	2	0	1	0	0	0	0	0	7 (4.1)
Linking papers	0	0	0	0	0	0	0	0	0	0	1	2	3 (1.8)
ICF only mentioned	0	0	0	0	0	1	0	0	0	0	1	0	2 (1.2)
Protocol	0	0	0	0	0	0	0	0	0	0	1	0	1 (0.6)
Review	0	0	1	2	0	1	0	3	2	1	5	6	21 (12.4)
Total	1	2	2	6	12	15	13	15	13	23	25	24	170 (100)

Disability and Rehabilitation and Journal of Rehabilitation Medicine published the majority of papers (35 (20.6%) and 17 (10.0%), respectively) and 64 papers (37.6%) were published in journals that just published one of the papers. Three authors were first authors of four of the included papers: Alguren, Paltamaa and Roe. Seven authors were first in three papers, 15 authors were first on two papers, and 107 authors were represented with one paper each.

The distribution between the five Nordic countries was diverse as more than half of the published papers were Swedish (55.9%), 42 (24.7%) papers from Norway, 24 (14.1%) papers from Finland, nine (5.3%) papers from Denmark and no papers from Iceland (Table 2).

**Table 2:** Related Countries for All Included Papers and Papers From the Two Categories “Clinical and/or Rehabilitation Contexts” and “Non-Clinical Contexts”

Country	Total papers (%)	“Clinical and/or rehabilitation contexts” and “non-clinical contexts” papers (%)
Sweden	96 (56.5)	53 (53.5)
Norway	42 (24.7)	26 (26.3)
Finland	23 (13.5)	18 (18.2)
Denmark	9 (5.3)	2 (2.0)
Iceland	0 (0)	0 (0)
Total	170 (100)	99 (100)

Table 3 shows area of focus in the included papers. The most frequently reported area of focus was neurology (25.9%), followed by musculoskeletal (18.2%), and work-related papers (10.0%). Area of focus was not relevant in 14 (8.2%) papers as they were editorials or reporting on ICF as a theoretical framework or in a general matter.

**Table 3:** Focus Areas for All Included Papers and Papers From the Two Categories “Clinical and/or Rehabilitation Contexts” and “Non-Clinical Contexts”

Focus area	Total papers (%)	“Clinical and/or rehabilitation contexts” and “non-clinical contexts” papers (%)
Neurology	44 (25.9)	27 (27.3)
Musculoskeletal	31 (18.2)	15 (15.2)
Work-related	17 (10.0)	17 (17.2)
Rheumatology	12 (7.1)	8 (8.1)
Mixed population	10 (5.9)	3 (3.0)
Community dwelling elderly	7 (4.1)	5 (5.1)
Psychiatry	7 (4.1)	4 (4.0)
Hearing loss	6 (3.5)	3 (3.0)
Heart/lung problems	3 (1.8)	3 (3.0)
Trauma	3 (1.8)	3 (3.0)
Cancer	3 (1.8)	1 (1.0)
Pain	2 (1.2)	2 (2.0)
Torture survivors	2 (1.2)	1 (1.0)
Other*	9 (5.3)	6 (6.1)
Not relevant†	14 (8.2)	1 (1.0)
Total	170 (100)	99 (100)

*Other: oral health, hemophilia, dysphagia, wheelchair users, leg ulcer patients, aphasia, young adults with a disability, education and alcohol dependency. †Not relevant is papers dealing with ICF framework.

In 102 papers (60.0%), a quantitative research method was used, 27 (15.9%) papers used qualitative research methods, and both methods were used in four (2.4%) of the papers; in 37 (21.8%) papers, this was not coded as the papers were reviews or editorials (29 papers (17.1%)) or “not relevant” to code (eight papers (4.7%)); the latter were papers describing development of ICF and ICF-related instruments.

###  “Clinical and/or rehabilitation contexts” and “non-clinical context”

Of the 170 selected papers, 99 were included in the two categories “clinical and/or rehabilitation contexts” and “non-clinical context”.

The publication years of the 99 papers follow the same pattern as the total 170 included papers (Table 1). A marked rise in papers from “clinical and/or rehabilitation contexts” and “non-clinical context” was observed in the last 3 years of the inclusion period.

Disability and Rehabilitation was the most frequently seen journal, publishing 24 (24.2%) of the papers. Journal of Rehabilitation Medicine published 11 (11.1%); 36 papers (36.4%) were published in journals that just published one paper each.

Four authors were first authors in three papers: Alguren [12-14], Paltamaa [15-17], Andelic [18-20] and Moller [21-23]. Eleven authors were first in two papers and 65 authors were each represented with one paper as first author.

The distribution between the five Nordic countries followed the distribution of the total 170 included papers (Table 2). Table 3 shows area of focus in the two categories. There were just minor differences between all included papers and papers from the category “clinical and/or rehabilitation contexts” and “non-clinical context”.

The most frequently reported area of focus was neurology 27 (27.3%), followed by work-related papers 17 (17.2%), and musculoskeletal 15 (15.2%). Area of focus was not relevant in one (1.0%) paper as it was on ICF as a general matter. In 73 papers (73.7%), a quantitative research method was used, 22 (22.2%) papers used qualitative research methods, and both methods were used in three (3.0%) of the papers; in one (1.0%) paper, this was not coded as the paper was “not relevant” to code because it described the development of an ICF-related instrument. Analysis on the five ICF components was performed: all five components were mentioned in results and/or discussion in 27 (27.3%) of the papers, whereas 11 (11.1%) papers mentioned none of the five components (Table 4). The component “activity” was the most frequently reported, appearing in 85 (85.9%) papers, and the component “personal factors”, not classified in ICF but relevant for the model, was the least frequently reported, being mentioned in 40 (40.4%) papers (Table 5).

**Table 4:** Number of ICF Components Used in Papers From the Two Categories “Clinical and/or Rehabilitation Contexts” and “Non-Clinical Contexts”

Components*	Papers (%)
0	11 (11.1)
1	5 (5.1)
2	9 (9.1)
3	18 (18.2)
4	29 (29.3)
5	27 (27.3)
Total	99 (100)

*Components (body functions, body structures, activity, participation, environmental factors, and personal factors) mentioned in results or discussion.

**Table 5:** Type of ICF Component Used in Papers From the Two Categories “Clinical and/or Rehabilitation Contexts” and “Non-Clinical Contexts”

Components*	Papers (%)
Body functions/body structures	68 (68.7)
Activity	85 (85.9)
Participation	79 (79.8)
Personal factors	40 (40.4)
Environmental factors	56 (56.6)

n = 99. *Components (body functions, body structures, activity, participation, environmental factors, and personal factors) mentioned in results or discussion.

Intervention was featured in 15 (15.1%) of the 99 papers in the category “clinical and/or rehabilitation contexts” [24-38]. One paper described an intervention for the staff and was therefore excluded from this group [39]. The areas of focus in the remaining 14 included papers describing intervention were: neurology four (28.6%) [25, 27, 30, 35]; musculoskeletal four (28.6%) [26, 28, 34, 38]; work-related three (21.4%) [29, 32, 36, 40]; heart/lung disorder one (7.1%) [37]; torture survivors one (7.1%) [24]; and other (aphasia) one (7.1%) [33]. Multidisciplinary intervention was the most frequent intervention as six papers (46.2%) described this type of intervention. In five of the six papers, a physician was involved amongst 3 - 4 other health professionals and social workers. The mono-disciplinary interventions were performed by medical doctors two (15.4%); occupational therapists one (7.7%); physical therapists three (23.1%); or logopedics one (7.7%). Six papers (42.9%) describing interventions were published during the last 3 years of the inclusion period. The distribution of papers presenting intervention studies between the Nordic countries was similar to the total sample of papers.

### Reviews

Twenty-one of the included papers were reviews [31, 41-60], focusing on the following areas of focus: musculoskeletal six (28.6%); neurology three (14.3%); rheumatology three (14.3%); mixed population three (14.3%); cancer two (9.5%); hearing loss one (4.8%); psychiatry one (4.8%); community dwelling elderly one (7.1%); and other (alcohol dependency) one (7.1%). More than half of the reviews (12 papers (57.1%)) were published after 2010. The distribution of reviews between the Nordic countries was similar to the distribution of other papers in the present review.

## Discussion

The present review’s objective was to quantify the development in utilization of ICF in the categories “clinical and/or rehabilitation contexts” and “non-clinical context”, and we found that this group was the largest with around 60% of all papers and there was a marked increase in 2005 - 2006 and after 2010.

Thus, this review confirms that the international trend of increased use of ICF is present in the Nordic countries too. Throughout the period under study (2001 - 2013), there has been a steady rise in the number of published ICF-related papers reflecting an increase in scientific papers in general. The results from the present review are in close accordance with the former review [6], although an exact comparison is not possible, because the exact number of papers from each country was not reported. Cerniauskaite et al reported 35 Swedish papers in the period 2001 - 2009; during the same period, almost the same number of published papers from Sweden [36] was found [6]. Jelsma found only 24 papers from the Nordic countries during the period 2001 - 2007, whilst the present review found 42 papers [7], although they represented a convenience sample and not all papers published up until 2007. Papers reporting ICF-CY or with persons aged < 18 as population were excluded, which might hamper comparison to the study by Jelsma.

The present review shows that the use of ICF is increasing, but there is a marked difference between the five Nordic countries. Norway, Finland and Denmark have almost the same population size, while the population of Sweden is twice as big. Norway and Sweden have the same rate of papers per inhabitant, Finland half that, Denmark just one-fifth, and no papers were included from Iceland, the smallest country.

This difference in number of papers might be explained by the fact that there has been heightened focus on rehabilitation in Norway by the government and the municipalities, and Sweden has a substantially higher number of academic therapists and nurses. Denmark has no medical speciality in rehabilitation and only few academic therapists and nurses; this might reduce the focus on the bio-psycho-social approach and thereby on the use of ICF.

The majority of the papers are published primarily in two rehabilitation journals which are promoting the ICF, and more than one-third is from other journals publishing only one paper. Although it is not possible to draw a firm conclusion from this review, these results do not support the goal for ICF to be the common language in function and disability.

When discussing the dissemination of ICF in clinical and rehabilitation research settings, one also has to consider the fact that ICF might not always be the right tool. The substantial core sets can prove too extensive in some types of data collection settings, and in cases where assessment of progression is a primary aim, ICF might not be the first choice. In other words, promoting ICF as the primary tool for evaluation of research in clinical and rehabilitation settings should be done recognizing such facts. One of the key elements in modern rehabilitation and the bio-psycho-social mind set is the context’s impact on function. The results from this systematic review do not fully support this, as we found the contextual factors were the least used components. It is also worth noting that a quarter of the papers mention just two or fewer components, and that only a quarter mentioned all five components. If the understanding of functioning as described in the ICF were to be fully implemented, more papers would have used all five components in at least the discussion of their results.

Regarding the distribution of categories, the present review found similar results to Cerniauskaite et al [6], whereas Jelsma did not report on the same categories [7]. Jelsma reported “specific health problems”, and as opposed to our results, she found that the musculoskeletal area was the most reported [7]. The former review did not report the total distribution of areas of focus, but found stroke and multiple sclerosis to be the most reported health conditions in “clinical or rehabilitation context” [6], which is similar to the present findings, where neurology was the most reported area of focus (26.3%). Thus, neurology or musculoskeletal issues as area of focus comprised the majority of papers. In both areas, the interdisciplinary approach is well known, and the bio-psycho-social approach that ICF offers might be easier implemented in these areas. In clinical, non-clinical and/or rehabilitation context, no papers from cancer research were found, and only few papers from COPD research. Despite that these diseases are known to hamper function, this might be due to the fact that these fields still have the greatest focus on body functions in rehabilitation. An increase in number of papers does not necessarily imply increase in quality, the present review performed no quality assessment or evaluations of the papers. Future research should evaluate whether the observed increase in quantity is seconded by a similar increase in quality. For ICF to be a practically applicable tool within clinical and rehabilitation settings, high-quality research is needed.

### Clinical messages

This review confirms the use of ICF in rehabilitation literature in the geographic selection “The Nordic countries”.

There is a marked difference in the use of ICF in different diagnosis and areas of focus.

The five ICF components are not systematically assessed in all ICF papers. However, at least four ICF components were mentioned in result or discussion in most papers, with the activity component being the most frequently reported.

One of the aims of the ICF was to provide a common language in rehabilitation. This aim is still not quite met and this review concludes: “There is still a long way to go before ICF is widely implemented in rehabilitation and clinical settings”.

### Limitations

This study was subject to four main limitations. First, the literature search included three databases: EMBASE, MEDLINE and PsycInfo, whilst other databases such as SCOPUS and CINAHL were excluded with potentially other papers. However, the methods used were largely based on the former review by Cerniauskaite et al [6], and as the aim was to compare the results, the search was limited to the same databases. Second, only English articles were selected; the literature search did not reveal papers in other languages. However, if other Nordic national databases had been included, more papers could be expected but the aim was to search internationally relevant papers. Thirdly, the review was performed by different teams of reviewers; the 1,623 identified records were initially screened by five review teams comprised of two persons each. This might hamper the reliability of the screening, as the different teams may have assessed the abstracts in the paper selection differently. In order to overcome this, all the reviewers screened a sample of 50 records, followed by a discussion of criteria for inclusion and exclusion. In case of doubt amongst the review teams, this was to be solved by the research group; this only was needed in two of the 1,623 records. The second reading of the included papers prompted discussion on another few papers, mostly regarding the area of focus; e.g. in some of the work-related papers as they focused on both work-related and neurological disease. This was solved by categorizing all work-related papers in this category, although the target population also belonged to one of the other categories. Finally, papers on ICF-CY or addressed persons aged < 18 years only were excluded. Including this category would potentially have added another 51 papers to the present review. This was to make the results comparable to the study by Cerniauskaite et al [6]; however, as mentioned before, it hampered the comparison to the study by Jelsma [7].

## Conclusion

In conclusion, this review systemically searched the ICF literature in the five Nordic countries with emphasis on the categories “clinical and/or rehabilitation contexts” and “non-clinical contexts” and found an increase in papers during the study period, especially in these categories. Sweden, Norway and Finland accounted for most papers, and in all papers the most represented focus areas were neurology, musculoskeletal, and work-related. Finally, in most papers, all five or at least four ICF components were mentioned in the result or discussion, with the activity component as the most frequently reported.

The answer to the question whether ICF provides a common language is mostly no. There is still a long way to go, before ICF is widely implemented in rehabilitation and clinical settings. The question is, whether the ICF, besides being a framework, also works as a classification system that supports standardized identification and description of health and health-related function. Further studies should be conducted in order to answer this question.

## References

[1] (2001). World Health Organization. ICF: International classification of functioning, disability and health. Geneva: WHO.

[2] Almborg AH, Welmer AK (2012). Use of the International Classification of Functioning, Disability and Health (ICF) in social services for elderly in Sweden. Disabil Rehabil.

[3] Maribo T, Melchiorsen H, Rubak DB, Jespersen E, Nielsen CV (2014). [Rehabilitation based on the bio-psychosocial model concerns health condition, functioning and contextual factors.]. Ugeskr Laeger.

[4] Stucki G, Reinhardt JD, Grimby G, Melvin J (2007). Developing "Human Functioning and Rehabilitation Research" from the comprehensive perspective. J Rehabil Med.

[5] Wade DT, Halligan P (2003). New wine in old bottles: the WHO ICF as an explanatory model of human behaviour. Clin Rehabil.

[6] Cerniauskaite M, Quintas R, Boldt C, Raggi A, Cieza A, Bickenbach JE, Leonardi M (2011). Systematic literature review on ICF from 2001 to 2009: its use, implementation and operationalisation. Disabil Rehabil.

[7] Jelsma J (2009). Use of the International Classification of Functioning, Disability and Health: a literature survey. J Rehabil Med.

[8] Imrie R (2004). Demystifying disability: a review of the International Classification of Functioning, Disability and Health. Sociol Health Illn.

[9] Madden RH, Dune T, Lukersmith S, Hartley S, Kuipers P, Gargett A, Llewellyn G (2014). The relevance of the International Classification of Functioning, Disability and Health (ICF) in monitoring and evaluating Community-based Rehabilitation (CBR). Disabil Rehabil.

[10] Saleeby P, Sykes C, Martinuzzi A, Hough J, Lee H, Leonardi M (2014). Developing Criteria to Evaluate ICF Literature. WHO - Family of International Classifications Network Annual Meeting.

[11] Kloda LA, Bartlett JC (2014). A characterization of clinical questions asked by rehabilitation therapists. J Med Libr Assoc.

[12] Alguren B, Bostan C, Christensson L, Fridlund B, Cieza A (2011). A multidisciplinary cross-cultural measurement of functioning after stroke: Rasch analysis of the brief ICF Core Set for stroke. Top Stroke Rehabil.

[13] Alguren B, Fridlund B, Cieza A, Sunnerhagen KS, Christensson L (2012). Factors associated with health-related quality of life after stroke: a 1-year prospective cohort study. Neurorehabil Neural Repair.

[14] Alguren B, Lundgren-Nilsson A, Sunnerhagen KS (2009). Facilitators and barriers of stroke survivors in the early post-stroke phase. Disabil Rehabil.

[15] Paltamaa J, Sarasoja T, Leskinen E, Wikstrom J, Malkia E (2008). Measuring deterioration in international classification of functioning domains of people with multiple sclerosis who are ambulatory. Phys Ther.

[16] Paltamaa J, Sarasoja T, Leskinen E, Wikstrom J, Malkia E (2007). Measures of physical functioning predict self-reported performance in self-care, mobility, and domestic life in ambulatory persons with multiple sclerosis. Arch Phys Med Rehabil.

[17] Paltamaa J, West H, Sarasoja T, Wikstrom J, Malkia E (2005). Reliability of physical functioning measures in ambulatory subjects with MS. Physiother Res Int.

[18] Andelic N, Johansen JB, Bautz-Holter E, Mengshoel AM, Bakke E, Roe C (2012). Linking self-determined functional problems of patients with neck pain to the International Classification of Functioning, Disability, and Health (ICF). Patient Prefer Adherence.

[19] Andelic N, Sigurdardottir S, Schanke AK, Sandvik L, Sveen U, Roe C (2010). Disability, physical health and mental health 1 year after traumatic brain injury. Disabil Rehabil.

[20] Andelic N, Stevens LF, Sigurdardottir S, Arango-Lasprilla JC, Roe C (2012). Associations between disability and employment 1 year after traumatic brain injury in a working age population. Brain Inj.

[21] Moller K (2003). Deafblindness: a challenge for assessment--is the ICF a useful tool?. Int J Audiol.

[22] Moller K, Eriksson K, Sadeghi AM, Moller C, Danermark B (2009). Long-term ophthalmic health care in Usher syndrome type I from an ICF perspective. Disabil Rehabil.

[23] Moller K, Danermark B (2007). Social recognition, participation, and the dynamic between the environment and personal factors of students with deafblindness. Am Ann Deaf.

[24] Agger I, Raghuvanshi L, Shabana S, Polatin P, Laursen LK (2009). Testimonial therapy. A pilot project to improve psychological wellbeing among survivors of torture in India. Torture.

[25] Bergemalm P, Borg E (2005). Peripheral and central audiological sequelae of closed head injury: Function, activity, participation and quality of life. Audiological Medicine.

[26] Fitinghoff H, Lindqvist B, Nygard L, Ekholm J, Schult ML (2011). The ICF and postsurgery occupational therapy after traumatic hand injury. Int J Rehabil Res.

[27] Hammer AM, Lindmark B (2010). Responsiveness and validity of the Motor Activity Log in patients during the subacute phase after stroke. Disabil Rehabil.

[28] Josephson I, Bulow P, Hedberg B (2011). Physiotherapists' clinical reasoning about patients with non-specific low back pain, as described by the International Classification of Functioning, Disability and Health. Disabil Rehabil.

[29] Nyberg VE, Novo M, Sjolund BH (2011). Do Multidimensional Pain Inventory scale score changes indicate risk of receiving sick leave benefits 1 year after a pain rehabilitation programme?. Disabil Rehabil.

[30] Pettersson I, Tornquist K, Ahlstrom G (2006). The effect of an outdoor powered wheelchair on activity and participation in users with stroke. Disabil Rehabil Assist Technol.

[31] Pless M, Granlund M (2012). Implementation of the International Classification of Functioning, Disability and Health (ICF) and the ICF Children and Youth Version (ICF-CY) within the context of augmentative and alternative communication. Augment Altern Commun.

[32] Puolakka K, Kautiainen H, Mottonen T, Hannonen P, Korpela M, Hakala M, Viikari-Juntura E (2009). A mismatch between self-reported physical work load and the HAQ: early identification of rheumatoid arthritis patients at risk for loss of work productivity. Clin Exp Rheumatol.

[33] Rautakoski P (2012). Self-perceptions of functional communication performance during total communication intervention. Aphasiology.

[34] Saltychev M, Kinnunen A, Laimi K (2013). Vocational rehabilitation evaluation and the International Classification of Functioning, Disability, and Health (ICF). J Occup Rehabil.

[35] Saltychev M, Tarvonen-Schroder S, Eskola M, Laimi K (2013). Selecting an optimal abbreviated ICF set for clinical practice among rehabilitants with subacute stroke: retrospective analysis of patient records. Int J Rehabil Res.

[36] Schult ML, Ekholm J (2006). Agreement of a work-capacity assessment with the World Health Organisation International Classification of Functioning, Disability and Health pain sets and back-to-work predictors. Int J Rehabil Res.

[37] Skumlien S, Skogedal EA, Bjortuft O, Ryg MS (2007). Four weeks' intensive rehabilitation generates significant health effects in COPD patients. Chron Respir Dis.

[38] Vaarbakken K, Ljunggren AE (2007). Superior effect of forceful compared with standard traction mobilizations in hip disability?. Adv Physiother.

[39] Pless M, Ibragimova N, Adolfsson M, Bjorck-Akesson E, Granlund M (2009). Evaluation of in-service training in using the ICF and ICF version for children and youth. J Rehabil Med.

[40] Puolakka K, Kautiainen H, Pekurinen M, Mottonen T, Hannonen P, Korpela M, Hakala M (2006). Monetary value of lost productivity over a five year follow up in early rheumatoid arthritis estimated on the basis of official register data on patients' sickness absence and gross income: experience from the FIN-RACo trial. Ann Rheum Dis.

[41] Aas RW, Tuntland H, Holte KA, Roe C, Lund T, Marklund S, Moller A (2011). Workplace interventions for neck pain in workers. Cochrane Database Syst Rev.

[42] Areskoug-Josefsson K, Oberg U (2009). A literature review of the sexual health of women with rheumatoid arthritis. Musculoskeletal Care.

[43] Blomdahl C, Gunnarsson AB, Guregard S, Bjorklund A (2013). A realist review of art therapy for clients with depression. Arts in Psychotherapy.

[44] Comins JD, Krogsgaard MR, Brodersen J (2013). Ensuring face validity in patient-related outcome scores--a matter of content. Knee.

[45] Eghdam A, Scholl J, Bartfai A, Koch S (2012). Information and communication technology to support self-management of patients with mild acquired cognitive impairments: systematic review. J Med Internet Res.

[46] Granberg S, Moller K, Skagerstrand A, Moller C, Danermark B (2014). The ICF Core Sets for hearing loss: researcher perspective, Part II: Linking outcome measures to the International Classification of Functioning, Disability and Health (ICF). Int J Audiol.

[47] Grotle M, Brox JI, Vollestad NK (2005). Functional status and disability questionnaires: what do they assess? A systematic review of back-specific outcome questionnaires. Spine (Phila Pa 1976).

[48] Gummesson C, Atroshi I, Ekdahl C (2004). The quality of reporting and outcome measures in randomized clinical trials related to upper-extremity disorders. J Hand Surg Am.

[49] Gustafsson S, Edberg AK, Johansson B, Dahlin-Ivanoff S (2009). Multi-component health promotion and disease prevention for community-dwelling frail elderly persons: a systematic review. European Journal of Ageing.

[50] Helbostad JL, Holen JC, Jordhoy MS, Ringdal GI, Oldervoll L, Kaasa S (2009). A first step in the development of an international self-report instrument for physical functioning in palliative cancer care: a systematic literature review and an expert opinion evaluation study. J Pain Symptom Manage.

[51] Jordhoy MS, Inger Ringdal G, Helbostad JL, Oldervoll L, Loge JH, Kaasa S (2007). Assessing physical functioning: a systematic review of quality of life measures developed for use in palliative care. Palliat Med.

[52] Levola J, Kaskela T, Holopainen A, Sabariego C, Tourunen J, Cieza A, Pitkanen T (2014). Psychosocial difficulties in alcohol dependence: a systematic review of activity limitations and participation restrictions. Disabil Rehabil.

[53] Lindner HY, Natterlund BS, Hermansson LM (2010). Upper limb prosthetic outcome measures: review and content comparison based on International Classification of Functioning, Disability and Health. Prosthet Orthot Int.

[54] Paltamaa J, Sjogren T, Peurala SH, Heinonen A (2012). Effects of physiotherapy interventions on balance in multiple sclerosis: a systematic review and meta-analysis of randomized controlled trials. J Rehabil Med.

[55] Petersson IF (2005). Evolution of team care and evaluation of effectiveness. Curr Opin Rheumatol.

[56] Pettersson I, Pettersson V, Frisk M (2012). ICF from an occupational therapy perspective in adult care: an integrative literature review. Scand J Occup Ther.

[57] Peurala SH, Kantanen MP, Sjogren T, Paltamaa J, Karhula M, Heinonen A (2012). Effectiveness of constraint-induced movement therapy on activity and participation after stroke: a systematic review and meta-analysis of randomized controlled trials. Clin Rehabil.

[58] Roe Y, Soberg HL, Bautz-Holter E, Ostensjo S (2013). A systematic review of measures of shoulder pain and functioning using the International classification of functioning, disability and health (ICF). BMC Musculoskelet Disord.

[59] Saltychev M, Eskola M, Tenovuo O, Laimi K (2013). Return to work after traumatic brain injury: Systematic review. Brain Inj.

[60] Vessby K, Kjellberg A (2010). Participation in occupational therapy research: a literature review. British Journal of Occupational Therapy.

